# Identification of differential expression genes related to anthocyanin biosynthesis in carmine radish (*Raphanus sativus* L.) fleshy roots using comparative RNA-Seq method

**DOI:** 10.1371/journal.pone.0231729

**Published:** 2020-04-24

**Authors:** Jian Gao, Wen-Bo Li, Hong-Fang Liu, Fa-Bo Chen

**Affiliations:** 1 School of Advanced Agriculture and Bioengineering, Yangtze Normal University, Chongqing, China; 2 Green Intelligence Environmental School, Yangtze Normal University, Fuling, China; ICAR-Indian Institute of Agricultural Biotechnology, INDIA

## Abstract

Radish (*Raphanus sativus* L.), is an important root vegetable crop grown worldwide, and it contains phyto-anthocyanins. However, only limited studies have been conducted to elucidate the molecular mechanisms underlying anthocyanin biosynthesis in the different color variants of the radish fleshy root. In this study, Illumina paired-end RNA-sequencing was employed to characterize the transcriptomic changes in seven different types of radish fleshy roots. Approximately, 126 co-modulated differentially expressed genes were obtained, and most DEGs were more likely to participate in anthocyanin biosynthesis, including two transcription factors *RsMYB_9* and *RsERF070*, and four functional genes *RsBRICK1*, *RsBRI1-like2*, *RsCOX1*, and *RsCRK10*. In addition, some related genes such as *RsCHS*, *RsCHI*, *RsANS*, *RsMT2-4*, *RsUF3GT*, glutathione S-transferase F12, *RsUFGT78D2-like* and *RsUDGT-75C1-like* significantly contributed to the regulatory mechanism of anthocyanin biosynthesis in the radish cultivars. Furthermore, gene ontology analysis revealed that the anthocyanin-containing compound biosynthetic process, anthocyanin-containing compound metabolic process, and significantly enriched pathways of the co-modulated DEGs were overrepresented in these cultivars. These results will expand our understanding of the complex molecular mechanism underlying anthocyanin synthesis-related genes in radish.

## Introduction

Anthocyanins, recognized as regulators of red to purple colors in nature, produce water-soluble pigments belonging to the flavonoid group [1]. Most studies conducted worldwide have demonstrated that anthocyanins, as a beneficial food additive, could help alleviate major public health concerns that include cardiovascular disease, inflammation, obesity, and diabetes, which are typically caused by consuming chemically synthesized food additives [2,3]. In addition, most of the regulatory genes have been found to be extensively involved in anthocyanin biosynthetic pathway, which are largely conserved in flowering plants [4]. Previous studies have demonstrated that anthocyanins are initially formed from phenylalanine by a series of enzymes involved in phenylpropanoid metabolism, such as phenylalanine ammonia-lyase (*PAL*), cinnamic 4-hydroxylase (*C4H*) and 4-coumarate-CoA ligase (*4CL*). This is subsequently followed by the action of chalcone synthase (*CHS*), and then the product 4, 2′4′6′-tetrahydrocychalcone, which are further catalyzed successively by four enzymes [Chalcone Isomerase (*CHI*), flavanone 3-Hydroxylase (*F3H*), dihydroflavonol 4-Reductase (*DFR*), as well as anthocyanidin synthase (*ANS/LDOX*)] [5,6]. However, the molecular mechanism of anthocyanin biosynthesis regulation is still not fully understood in the different color variants of radish fleshy roots.

Recently, based on the global transcriptome technology (such as RNA-Seq technology), most differentially expressed genes association with anthocyanin biosynthesis and the expression of anthocyanin biosynthesis have been identified in a majority of important fruit crops such as grape[7], blood orange [8] and blueberry [9]. However, transcriptome analysis of anthocyanin biosynthesis and the expression of anthocyanin biosynthesis related genes in ‘Hongxin’ radish, which is known for containing a natural red pigment (red radish pigment), and is produced in Chongqing City, have not been fully investigated.

Radish is a biennial root vegetable crop belonging to the family Brassicaceae. It is an economically important vegetable crop with an edible taproot. The ‘HX-1’ and ‘WG-1’ inbred lines, and their respective elite inbred lines ‘HX-2’, ‘HX-3,’ and ‘WG-2’, ‘WG-3’ with different colors of fleshy roots, were cultivated as outstanding local cultivars in Fuling, Chongqing. In the present study, the local cultivars ‘HX-1’ and ‘WG-1’ inbred lines, their respective elite inbred lines ‘HX-2’, ‘HX-3’ and ‘WG-2’, ‘WG-3’, and white skin and white flesh (WW) from white radish (NM) were used as the experimental materials to conduct RNA-Seq. Subsequently, the putative candidate genes involved in the biosynthesis of anthocyanins were identified. Next, differentially gene expression (DGE) profile analysis was used to identify the putative transcripts involved in the biosynthesis of anthocyanins that are found in six different radish fleshy root types, and were compared with WW_NM.

## Materials and methods

### Plant material and experiment design

**Fig 1:**
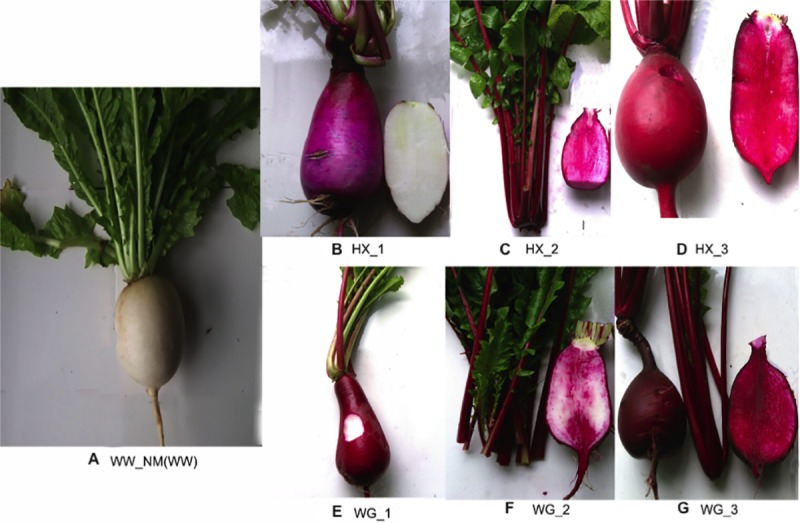
Fleshy roots from seven types of radish are shown in A-G, including WW_NM (white radish white skin and white flesh root, WW), HX-1 (Hongxin red skin and white flesh root, RW), HX-2 (Hongxin red skin and pinky flesh root, RP), HX-3 (Hongxin red skin and red flesh root, RR), WG-1 (Waguan red skin and white flesh root, RW), WG-2 (Waguan red skin and pinkly flesh root, RP), and WG-3 (Waguan red skin and red flesh root, RR) respectively.

The young fleshy roots obtained from seven differently colored radish variants (‘WW’, ‘HX-1’, ‘HX-2’, ‘HX-3’, ‘WG-1’, ‘WG-2’, and ‘WG-3’) were selected as experiment materials used for RNA-Seq in this study. (Fig 1, S1 Table). Of these genotypes, ‘WW’ represents white skin and white flesh root obtained from white radish. ‘HX-2’ and ‘HX-3’ represent elite inbred lines selected from inbred line ‘HX-1’ followed by genetic improvement for 5 generations, ‘WG-2’ and ‘WG-3’ represent elite inbred lines selected from inbred line ‘WG-1’ followed by genetic improvement for 5 generations. The cultivars exhibited, red skin and white fleshy root (RW), red skin and pink fleshy root (RP), and red skin and red fleshy root (RR) for ‘HX-1’, ‘HX-2’ and ‘HX-3’, as well as ‘WG-1’, ‘WG-2’ and ‘WG-3’, respectively. To identify the pigment contents of the seven colored radish variants, anthocyanidin profiles of fleshy roots from the seven inbred lines of radish were investigated through HPLC analysis (S1 Table). The fleshy roots were collected from three homozygous varieties of the seven inbred lines of radish and they were pooled together. The fleshy root tissues were grinded with liquid nitrogen. Next, we extracted the red pigment using a solvent mixture containing methanol (40%, v/v), formic acid (0.1%, v/v), and acetone (40%, v/v). We used 10 μL injection volumes for a VDS C-18 column (4.6 × 250 mm, 5 μm, VDS Optilab, Germany) with 0.8 mL/min flow rate.

Seven inbred lines of radish (‘WW’,’HX-1’,’HX-2’, ‘HX-3’, ‘WG-1’,’WG-2’, and ‘WG-3’) were used in this study. All the samples were cultivated in a greenhouse at the experimental farm of Yihe (Yangtze normal university experiment base) in 2018. First, we sowed the seeds of each inbred line in sterilized soil for 2 weeks under normal growth conditions (23°C, 16 h light/8 h dark). Next, the 2-week-old plants were transferred and kept for 15 days in a cold room (5 ± 1°C, 12 h light/12 h dark) for vernalization treatment. After the vernalization periods, the plants were grown in a normal growth room for 30 days under normal growth conditions (23°C, 16 h light/8 h dark). Additionally, for the current sample collection, we selected the bolting plants when the length of the floral axis was ≥ 1 cm for each radish inbred line experiment. Two independent biological replicates of each fleshy root sample were collected for RNA-Seq. All harvested tissues were immediately frozen in liquid nitrogen and stored at ˗80°C for RNA-Seq analysis.

### Sample preparation and library construction

The mRNAs were isolated by magnetic beads with Oligo(dT) and broken into short fragments by fragmentation buffer. Subsequently, the first strands of cDNA were synthesized using short fragments as templates and random hexamers as primers while the second strands of cDNA were synthesized by adding it into the buffer, dNTPs, RNase H, and DNA polymerase I. Next, the synthesized cDNA fragments were purified (QIAquick PCR kit) and resolved with EB buffer for end repair, single nucleotide A (adenine) addition, and connection of adapters. Lastly, the desired fragment was harvested from the recovery of agarose gel electrophoresis and amplified by PCR. Ultimately, the prepared library was sequenced with two replicates using Illumina HiSeq2000. Finally, raw data of trancriptome sequence was submitted to NCBI with the accession number of PRJNA613533.

### Reads processing and differentially expressed genes (DEGs) identification

After filtering out adaptor-only reads, trimming reads, and low-quality reads (base quality ≤ 10) using Trimmomatic with paramers “PE ILLUMINACLIP: merged_adapters.fa:2:30:10 LEADING:3 TRAILING:3 SLIDINGWINDOW:4:15 MINLEN:25”, we aligned the clean high-quality reads to the SSU and LSU rRNA sequences [10] using BWA software [11] with the following parameter: -n 4 -o 1 -e 1 -i 0 -l 50 -k 2. Subsequently, clean reads were then *de novo* assembled into transcripts using Trinity software. To obtain as much description as possible for the assembled sequences, all unigenes were annotated based on BLAST searches in the National Center for Biotechnology Information (NCBI) non-redundant protein (Nr) and Swiss-Pro protein databases using BLASTx search tool with threshold E-value set at 1e-10. Based on Nr and SwissPro BLAST results, the unigenes were then annotated in gene ontology (GO, http://www.geneontology.org/) and kyoto encyclopedia of genes and genomes (KEGG, http://www.kegg.jp/) databases to further predict their functions. Next, Bowtie2 was adopted to map the clean reads of seven different fleshy root libraries to the *de novo* assembled transcriptome with default parameters respectively, and the transcript abundances were assessed and carried out with RSEM (RNA-Seq by Expectation Maximization) was launched using the scripts provided in the Trinity pipeline through transcript quantification of the *de novo* assembly with default parameters. To generate a list of genes with significant base level expression and fewer false positives than a lower expression level threshold, only transcripts FPKM ≥ 1 were considered to be significantly expressed in this study [12]. To quantify the reproducibility of data for the biological replicates of seven radish fleshy root types, analysis of Spearman’s correlation coefficient (SCC) that calculated the log10-transformed FPKM values of the expressed genes was conducted by the Cor.test functions in R. After calculating gene expression levels, DEGs were screened by noiseqbio [13] and were identified using a corrected *P*-value < 0.05 between each set of compared samples. The fold change of the gene expression of six cultivars of radish comprising of ‘HX-1’, ‘HX-2’, ‘HX-3’, ‘WG-1’, ‘WG-2’ and ‘WG-3 ‘ were identified by comparing with ‘WW _ NM’ respectively; for example ‘HX-1’ vs. ‘WW’, ‘HX-2’ vs. ‘WW’, ‘HX-3’ vs. ‘WW’, ‘WG-1’ vs. ‘WW’, ‘WG-2’ vs. ‘WW’, and ‘WG-3’ vs. ‘WW’. Furthermore, neighbor–joining cluster was used to analyze anthocyanin synthesis-related genes (ASRGs).

### GO functional annotation and KEGG pathway analysis of co-modulated DEGs in radish

Co-modulated DEGs (Common DEGs in both inbred lines ‘HX’ and ‘WG’) were identified using a venny graph. GO annotation and KEGG pathway enrichment of co-modulated DEGs were also conducted. GO functional categories were assigned to DEGs through GO database (http://www.geneontology.org/). Moreover, KEGG pathway enrichment analysis of DEGs was identified by testing the statistical enrichment using KOBAS software [14]. The co-modulated DEGs in Hongxin and Waguan were annotated to KEGG database; *p*-value and false discovery rate (FDR < 0.05) were presented after pathway analysis for each type. The degree of KEGG enrichment was evaluated as the rich factor, *q*-value, and the number of genes in the enriched pathway. The rich factor refers to the ratio of the number of DEGs to the number of total annotated genes in a certain pathway. The *q*-value is a multiple hypothesis-corrected *p*-value. The *q*-value consists of values between 0 and 1; values closer to 0 indicate a more significant enrichment. Next, R script was used to construct their relative graphs.

### Validation of candidate ASRGs in radish using real-time qRT-PCR

To confirm the results obtained from RNA-Seq assay, 15 anthocyanin synthesis related genes (ASRGs) were chosen and validated by qRT-PCR, based on their marked level of expression alteration. The primers were designed by Primer 5.0 software for qRT-PCR experiments and radish gene (Actin) was used as a standard control (**S2 Table**). The amplification programs were performed according to the standard protocol of ABI7500 system, and conducted in triplicates as mentioned by Gao et al.[15]. The relative quantitative method (2^-△△CT^) was used to calculate the fold change in the expression levels of the target genes [16].

## Results

### Illumina sequencing and *de novo* assembly

These seven RNA-Seq libraries generated over 351.4 million raw reads (two replicates) on the average. After quality filtering, 39.4 million clean reads were obtained in each library and the percentage of clean reads was almost 78% after removing the rRNA sequences among raw tags in each library (**S3 Table**). Moreover, 198,342 assembled transcripts from the clean reads were constructed with an average length of 411 bp, and 34,927 unigenes (N50 = 1621 bp) were generated with paired-end reads with an average length of 768 bp using *de novo* assembly technology, more than 69.2% of all unigenes ranged from 500 to 1500 bp were identified as the predominant length of the assembled unigenes (**S1A Fig**).

### Functional annotation and classification of unigenes in radish

We first aligned the assembled sequences to the public plant protein databases from NCBI-Nr, as well as Swiss-Prot protein databases through BLASTx, and GO terms for functional classification. In total, 28,758 significant BLAST hits were returned, and three main categories of GO classification comprising of biological process (BP), molecular function (MF), and cellular component (CC) were analyzed separately. The cellular (GO:0009987) and metabolic (GO:0008152) processes within BP, binding (GO:0005488) and catalytic (GO:0003824) activities within MF, and cells (GO:0005623) and organelles (GO:0043226) within CC were found as the most representative level 2 GO terms in all three data sets **(S1B Fig)**. Furthermore, all assembled radish unigenes were mapped to canonical pathways as the reference for KEGG pathway analysis. 14,107 unigenes were significantly matched in the KEGG database, and were assigned to 138 biosynthesis pathways classified into 19 subclass categories. The result showed that the largest pathway group was the translation pathway containing more than 3,000 members (unigene products), followed by environmental adaption, signal transduction, transportation, and catabolism pathways from 1,500 to 3,000 unigene products. Additionally, in these categories, membrane transport, glycan biosynthesis, and metabolism were the relatively small pathways, containing less than 500 members **(S1C Fig**).

### DEGs association with anthocyanin synthesis traits in both radish cultivars

**Fig 2:**
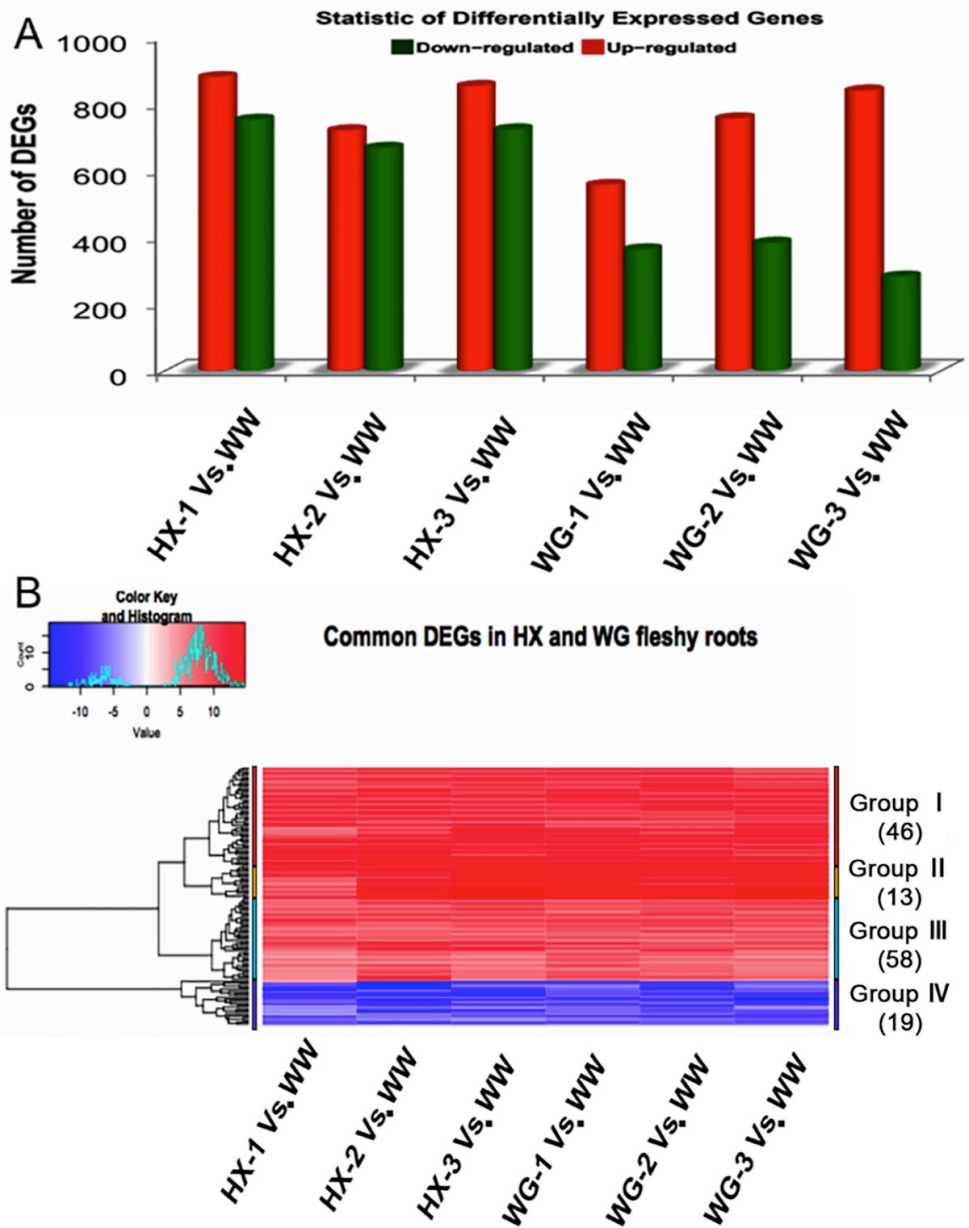
Transcriptional changes of anthocyanin synthesis-related genes in radish variant. (A) Statistics of differentially expressed genes (including up-regulated and down-regulated genes in each comparison groups) in seven radish color variants by performing pair-wise comparisons of the two radish cultivars ‘HX-1’ and ‘WG-1’, as well as their elite inbred lines ‘HX-2’, ‘HX-3’ and ‘WG-2’, ‘WG-3’. For each radish phenotype, comparison of changes in gene expression between NM_WW group and colored radish variant (‘HX-1’ and ‘WG-1’, as well as their elite inbred lines ‘HX-2’, ‘HX-3’ and ‘WG-2’, ‘WG-3’) was conducted. (B) Clustering and heat map of common differentially expressed genes (co-modulated genes) based on the expression profiles of the two radish cultivars ‘HX-1’ and ‘WG-1’, as well as their elite inbred lines ‘HX-2’, ‘HX-3’ and ‘WG-2’, ‘WG-3’.

In radish, to identify the putative candidate genes with marked changes and involved in anthocyanin biosynthesis, Bowtie2 was used to map the clean reads from the seven different fleshy root libraries to *de novo* assembly transcriptome reference sequences, and RSEM software was used to qualify the clear reads by assigning unigenes and isoforms to transcriptome [12]. The FPKM cutoff was set to 1 as the standard level for calculating the assigned unigene and isoform expression (**S4 Table**). We found that a higher SCC among biological replicates existed in our gene expression data, indicating that sequencing replicates exhibits high correlation between each group of samples (**S2 Fig**). Moreover, normalized expression levels were analyzed for all globally expressed genes, indicating that high distinct gene expression profiles exist (**S3 Fig**). Furthermore, DEGs were identified in all radish fleshy roots including different WW_NM groups and other radish groups (‘HX-1’ and ‘WG-1’, and their advanced inbred lines ‘HX-2’, ‘HX-3’ and ‘WG-2’, ‘WG-3’). These results indicated that 1,630, 1,386, and 1,574 DEGs were generated in HX-1, HX-2, and HX-3 respectively, compared to NM_WW, including up-regulated (879,719 and 852 transcripts) and down-regulated (751,667 and 722 transcripts) genes. In addition, 919, 1,138, and 1,120 DEGs were detected in WG-1, WG-2, and WG-3, respectively compared to WW_NM, including up-regulated (556, 755, and 838 transcripts) and down-regulated (363, 383, and 282 transcripts) genes (**Fig 2A**). Changes in expression pattern of co-modulated DEGs were displayed with different colors using heat maps. Interestingly, co-modulated DEGs showed similar expression trends in the fleshy roots of both the radish cultivars, including most of the up-regulated DEGs (**Fig 2B**); conserved DEGs were identified in both radish cultivars. These co-modulated DEGs were grouped into two classes based on the overall gene expression patterns. Candidate genes assigned to Groups I, II, and III denoted up-regulated expression trends in both radish cultivars, while genes in Group IV were down-regulated in Hongxin (HX) and Waguan (WG) radish, including metallothionein-like protein type 2, *MT24/MT2-25* (Cluster_102696 and Cluster_51879), methionine aminotransferase *BCAT4-like* (Cluster_18143), and dihydrolipoyl dehydrogenase 1, chloroplastic (Cluster_1747). In addition, some functional genes encoding anthocyanidin related proteins were up-regulated in Group II, followed by sets of transport proteins and transcription factors, such as protein *BRICK1* (Cluster_32957), glutathione S-transferase F12 (Cluster_24268), and *MYB* transcription factor (Cluster_46036) (**Fig 2B, S5 Table**).

### Functional annotation of genotype-specific DEGs and co-modulated DEGs association with anthocyanin synthesis traits

**Fig 3:**
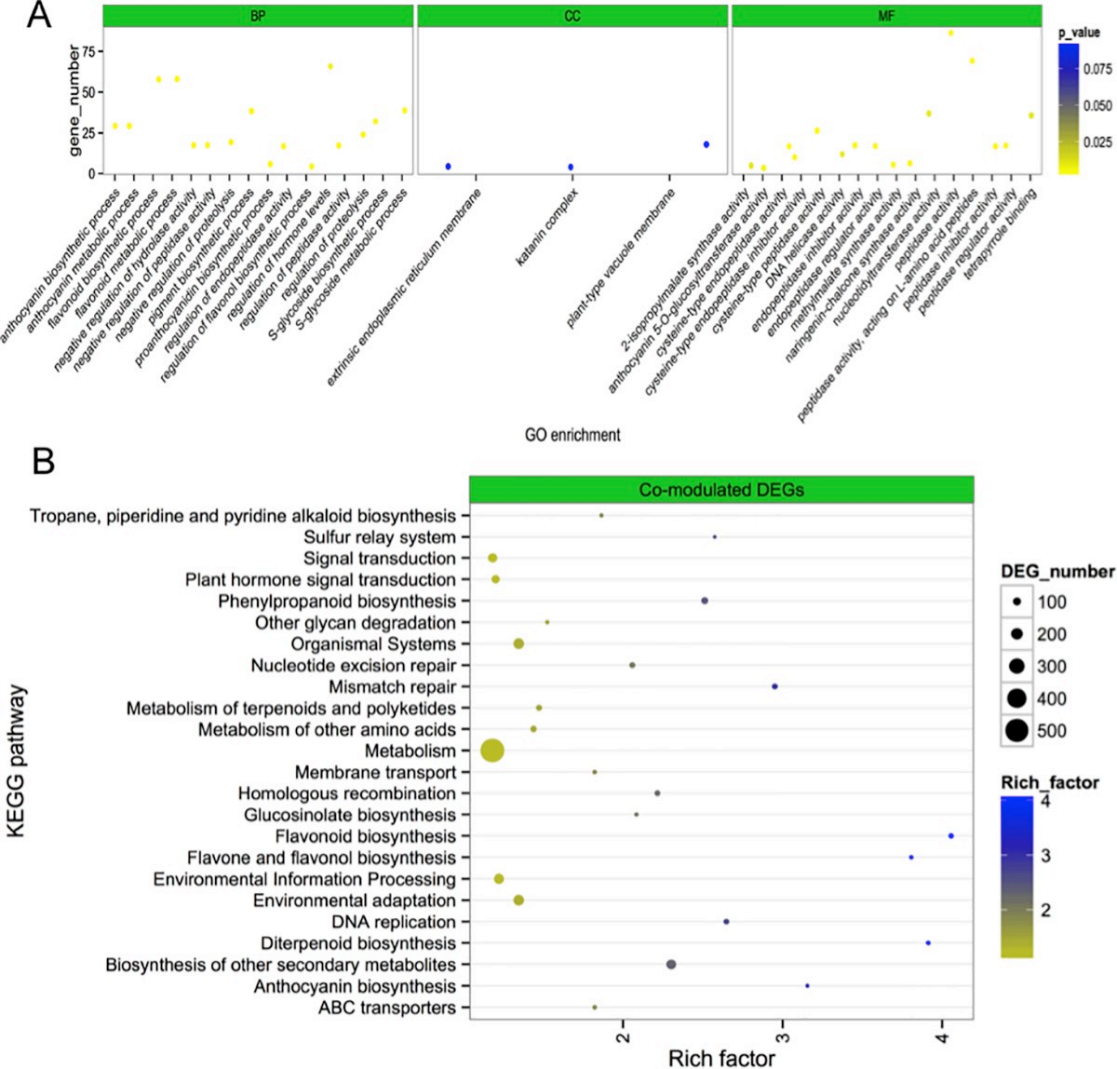
Functional enrichment analysis of differentially expressed genes (co-modulated DEGs) in radish. (A) Enriched GO terms of co-modulated differentially expressed genes related to anthocyanin synthesis in radish. (B) Pathway enrichment analysis of differentially expressed genes related to anthocyanin synthesis in radish. KEGG pathways were plotted on the ordinate, and the enrichment factor (rich factor) was plotted on the abscissa. The color of points represents the q-value, and the size of points represents the number of DEGs mapped to the reference pathway. Legends for the color scale of q-values and size-scaling of the number of DEGs are shown to the right of the plot.

To explore the regulatory mechanisms associated with anthocyanin synthesis traits, GO annotation, and KEGG pathway enrichment of co-modulated DEGs and transcripts for genotype-specific DEGs were evaluated. The results illustrated that GO terms comprising of “anthocyanin-containing compound biosynthetic process”, “anthocyanin-containing compound metabolic process,” and “flavonoid biosynthetic process” were overrepresented in all genotypes (**S6 Table, Fig 3A**). KOBAS software was used to validate the biological functions of co-modulated genes and genotype-specific DEGs by conducting pathway enrichment analysis. These results indicated that the five significantly enriched pathways for co-modulated DEGs were as follows: flavonoid, flavone, flavonol, diterpenoid, anthocyanin, and phenylpropanoid biosyntheses (**S7 Table, Fig 3B**).

### Validation of DEGs related to anthocyanin synthesis traits through qRT-PCR

**Fig 4:**
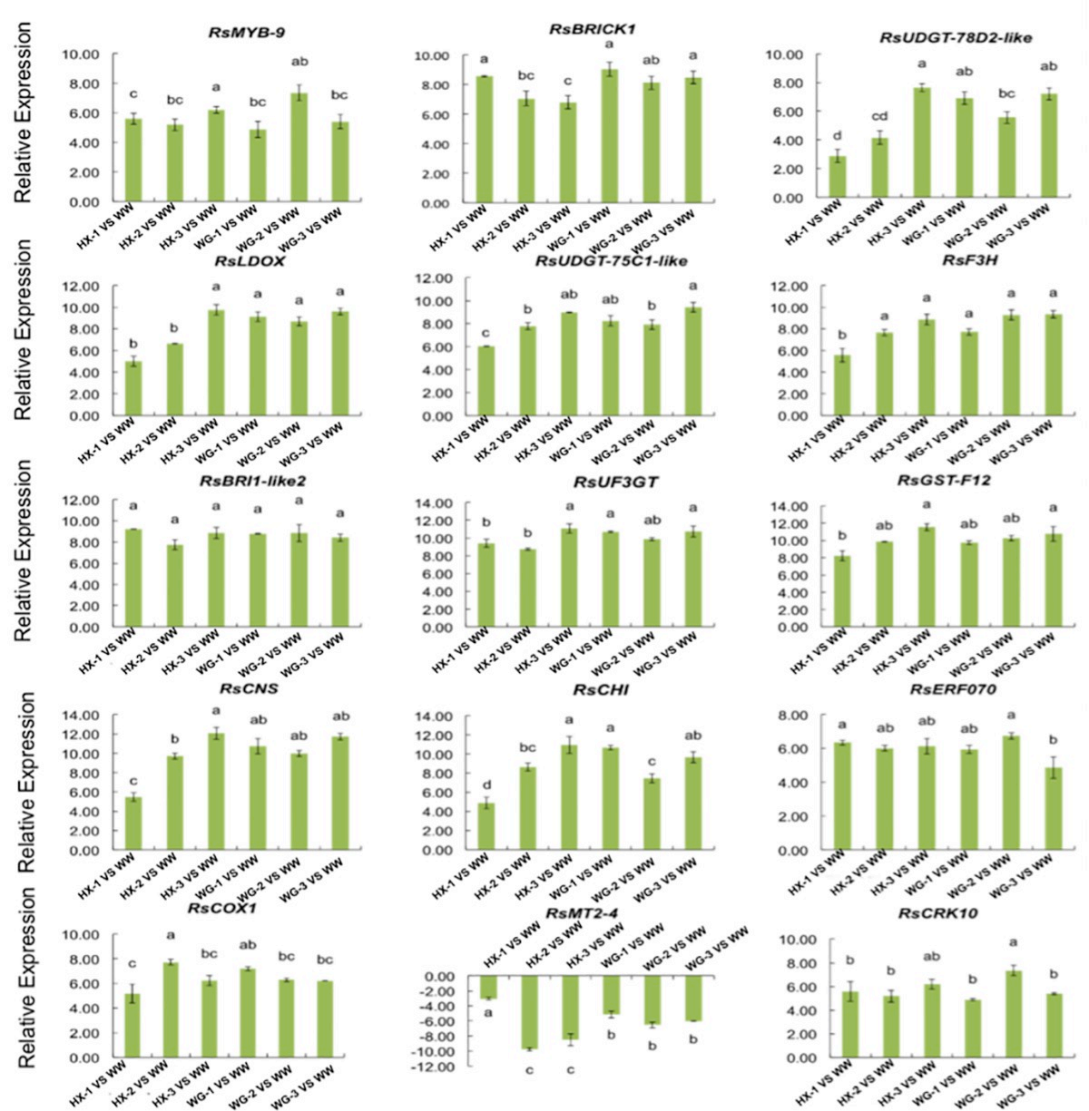
Transcriptional analysis of anthocyanin synthesis-related genes (ASRGs) identified in six cultivars of radish (‘HX-1’,’HX-2’,’HX-3’,’WG-1’,’WG-2’ and ‘WG-3’) using qRT-PCR, compared to WW_NM radish. Relative gene expression levels were normalized against actin transcript levels, and fold change of gene expression of six cultivars of radish comprising of ‘HX-1’, ‘HX-2’, ‘HX-3’, ‘WG-1’, ‘WG-2’ and ‘WG-3 ‘ was identified by comparing each cultivar with ‘WW_NM’, such as ‘HX-1’ vs. ‘WW’, ‘HX-2’ vs. ‘WW’, ‘HX-3’ vs. ‘WW’, ‘WG-1’ vs. ‘WW’, ‘WG-2’ vs. ‘WW’, and ‘WG-3’ vs. ‘WW’. The standard error calculated from three biological replicates and significant (P < 0.05) difference identified by uncorrected Fisher’s LSD test in multiple comparisons after two-way ANOVA are indicated by error bars and stars, respectively. Values connected by the same letter within the same gene and data collection point are not significantly different at P ≤ 0.05.

To experimentally confirm the DEGs obtained from sequencing and computational analysis based on GO annotation and KEGG pathway enrichment analysis, 15 putative DEGs related to anthocyanin synthesis (up-regulated expression of Cluster_46036, Cluster_32957, Cluster_11854, Cluster_3903, Cluster_2378, Cluster_4431, Cluster_11910, Cluster_9270, Cluster_24268, Cluster_29200, Cluster_23793, Cluster_25088, Cluster_10770, and Cluster_30537 and down-regulated expression of Cluster_51879) were subjected to qRT-PCR analysis. At last, 15 ASRGs with marked alteration in anthocyanin synthesis pathway were validated using qRT-PCR **(Fig 4, S8 Table**). The Person correlation analysis based on log2 fold change measurement of the 15 putative DEGs related to anthocyanin synthesis for the qRT-PCR analysis is higher between RNA-Seq and qRT-PCR (R2 = 0.86563), indicating excellent concordance between the two methods (**S4 Fig**).

Anthocyanins are formed through phenylpropanoid metabolism of phenylalanine by a series of enzymes. In this study, it was shown that the transcripts of *RsCHS (Cluster_29200)*, *RsCHI(Cluster_23793)*, *RsDFR(Cluster_3903)*, and *RsF3H(Cluster_4431)* were abundantly expressed in the root tissues of all genotypes, with the highest expression levels observed in advanced inbred lines ‘HX-3’ and ‘WG-3’ roots. The results showed that the expression levels of *RsCHS*, *RsCHI*, and *RsDFR* was more abundant in root of “HX-3” than root of “WG-3”, except for *RsF3H* (**Fig 4**). In addition, *RsMT2-4 (Cluster_51879)* and *RsUF3GT* (Cluster_9270) were involved in methylation of anthocyanidins, resulting in stable compounds. The transcripts of *RsMT2-4 (Cluster_51879)* were significantly down-regulated in the root of ‘HX-1’, ‘WG-1’, and their corresponding advanced inbred lines compared to the expression level of *RsMT2-4 (Cluster_51879)* in ‘WW’. In contrast, *RsUFGT78D2-like (Cluster_11854)* and *RsUDGT-75C1-like (Cluster_2378)* were consistently increased in ‘HX-1’ and their corresponding advance inbred lines compared to their expression levels in ‘WW’, but no significant increase in ‘WG-1’ and their corresponding advance inbred lines was observed (**Fig 4**). Generally, the transcripts of *RsUFGT78D2-like (Cluster_11854)* and *RsUDGT-75C1-like (Cluster_2378)* were up-regulated in colored radish cultivars and contained high anthocyanin content. Contrastingly, the transcripts of *RsMT2-4* were downgraded. In addition, glutathione S-transferase F12 (Cluster_24268) and *RsUF3GT* were significantly up-regulated in fleshy root types, occurring more abundantly in the root of “HX-3” than “WG-3”.

Transcription factors and functional proteins were also reported to be involved in anthocyanin biosynthesis. In this study, two transcription factors (Cluster_46036 encoded *RsMYB_9*, and Cluster_25088 encoded *RsERF070*) were experimentally validated by qRT-PCR. The results showed that the transcripts of *RsMYB_9* and *RsERF070* were elevated in colored radish cultivars with high anthocyanin content, but did not show significant expression when compared to each other, except for *RsMYB_9*, which showed highest expression in ‘HX-3’, and *RsERF070* that showed lowest expression in *‘*WG-3*’*. Moreover, four functional proteins comprising of *RsBRICK1* (Cluster_32957), *RsBRI1-like2* (Cluster_11910), *RsCOX1* (Cluster_10770) and *RsCRK10* (Cluster_30537) were significantly up-regulated in the different fleshy roots types compared to white radish. *RsBRI1-like2* protein (Cluster_11910) did not show significant expression changes when compared to each other. *RsCOX1* (Cluster_10770) showed the highest changes in ‘HX-2’; however, *RsERF070* showed the lowest expression in ‘HX-1*’* without significant expression in ‘WG-1’ and advanced lines. Moreover, *RsCRK10* (Cluster_30537) was found to be highly accumulated in ‘HX-2’, but *RsBRICK1* (Cluster_32957) was found to be less accumulated in ‘HX-3’.

## Discussion

Anthocyanins play diverse physiological roles in plants, and they have been reported as one of the major color pigments [17,18]. In this study, different anthocyanin synthesis-related traits of the fleshy roots were identified following *de novo* transcriptome analysis of seven different cultivars; approximately 34,927 unigenes were obtained in different cultivars. Additionally, 14,117 unigenes were successfully annotated through BLASTX search of KEGG database, and approximately 17,216 global expressed genes from sets of RNA-Seq experiments were identified. Moreover, many cultivars-specific transcriptional DEGs were observed, and 191 and 142 specific DEGs with marked alteration were identified to be associated with anthocyanin synthesis traits in HX and WG, respectively (**S5 Fig**). Of these DEGs, 126 were co-modulated in both cultivars associated with anthocyanin synthesis traits in the seven differential fleshy roots types, including ‘HX-1’, ‘HX-2’, ‘HX-3’, ‘WG-1’ ‘WG-2’, and ‘WG-3’. The expression of some functional genes encoding anthocyanidin related proteins was up-regulated in group II, along with a diverse set of transport proteins and transcription factors, such as protein *BRICK1* (Cluster_32957), glutathione S-transferase F12 (Cluster_24268), and *MYB* transcription factor (Cluster_46036) (**Fig 4**). To date, *GSTs* are involved in anthocyanin transport based on genetic and biochemical evidences [19]. *Bz2* was first demonstrated in *Zea mays* by its mutant bronze-2 as GST-encoding gene, which is involved in vacuolar transfer of anthocyanins (*bz2*) [20]. Anthocyanin accumulation and pigment mislocalization were reduced in Arabidopsis, caused by mutations in the *GST*-encoding genes [21].

In this study, *GST-F12* (Cluster_24268) was significantly up-regulated in different fleshy root types. These findings provided further evidences for the role of *GSH* in anthocyanin transport mechanisms. Moreover, *MYB* is a key component of the central regulatory mechanism to determine the variations in anthocyanin production [22]. In the current study, four anthocyanin biosynthesis-related genes comprising *CHS* (Cluster_29200 and Cluster_49669), *CHI* (Cluster_23793 and Cluster_85152), and *F3H* (Cluster_14857) were significantly up-regulated in both ‘HX-1’ and ‘WG-1’ radishes, as well as in their advanced inbred lines compared to NM_WW (**Fig 4**). Previous studies have shown that *CHS* could act as the key gene in flavonoid and anthocyanin pathways that was demonstrated by targeting the gene for silencing in strawberry [23]. Additionally, *F3H* has been demonstrated as the key regulator in anthocyanin synthesis using transient silencing. F3H-RNAi fruits showed reduced anthocyanin and flavonol contents [24]. In addition, a similar cloning approach was used to transiently silence *DFR* in strawberry [25]. In our study, *CHS* (Cluster_29200 and Cluster_49669), *CHI* (Cluster_23793 and Cluster_85152), *F3H* (Cluster_14857), and *DFR* (Cluster_13775) were dynamically up-regulated with marked positive and significant correlation with red pigment content in different fleshy roots types of HX (‘HX-1’, ‘HX-2’, ‘HX-3’) radish cultivars. However, this was not observed in different fleshy root types of WG (‘WG-1’ ‘WG-2’ and ‘WG-3’) radish; except for *CHS* encoded by Cluster_49669 unigene. We inferred that ‘HX-1’ radish and its advanced inbred lines might be an ideal radish model for anthocyanin synthesis-related genes, as well as for the study of molecular mechanism underlying anthocyanin synthesis pathway. More importantly, the roles of *MYB* transcription factors in the regulation of pigmentation in plants have been extensively studied [26]. It is known that R2R3-type MYB proteins and MYB-bHLH-WD40 complex directly activate the transcription of structural genes in anthocyanin pathway, such as transcription of early (*CHS*, *CHI*, *F3’H* and *FLS*) and late (*DFR*, *ANS* and *ANR*) flavonoid biosynthesis genes, respectively [27]. In this study, we also demonstrated that *MYB* transcription factors (Cluster_46036) were significantly and dynamically up-regulated, and showed marked positive and significant correlation to red pigment content in different fleshy root types of HX (HX-1, HX-2 and HX-3) radish, but not of WG radish (**S5 Table**). Therefore, we inferred that in HX radish, *MYB* transcription factors (Cluster_46036) may specifically activate early flavonoid biosynthesis genes such as *CHS* (Cluster_29200 and Cluster_90751), *CHI* (Cluster_23793 and Cluster_85152) and *F3’H* (Cluster_14857), as well as late flavonoid biosynthesis genes consisting of *DFR* (Cluster_93919 and Cluster_13775) and *ANS* (Cluster_15057 and Cluster_97432) in HX radish, thereby directly playing important roles in anthocyanin biosynthesis, for WG radish. Anthocyanin biosynthesis-related genes may be regulated by other means; however, their molecular regulation mechanism awaits further investigation.

## Supporting information

S1 Fig *De novo* assembly of radish transcriptome and functional classification of assembled unigenes from GO and KEGG analysis. A. Length distribution of contigs and unigenes from radish transcriptome, B. Histogram representation of GO terms. Unigenes were assigned to gene othology (GO) terms comprising “BP”, “MF,” and “CC” for functional classification. C. Pathway assignment based on the KEGG, Non-redundant unigenes for radish transcriptome were assigned into 138 biosynthesis pathways and classified into 19 subclass categories.
(TIF)

S2 Fig SCC analysis of mRNA data for the seven different fleshy root libraries (NM_WW, HX-1, HX-2, HX-3, WG-1, WG-2 and WG-3) using log10-based FPKM values. The hierarchical clustering dendrogram was inferred according to SCC analysis result.
(TIF)

S3 Fig Normalized expression levels for all globally expressed genes showed gene expression profiles in seven different fleshy root libraries (NM_WW, HX-1, HX-2, HX-3, WG-1, WG-2, and WG-3) S4 Fig: Venn diagram analysis of common anthocyanin synthesis related genes in seven different fleshy root libraries (NM_WW, HX-1, HX-2, HX-3, WG-1, WG-2 and WG-3) (HX-1 vs. NM_WW, HX-2 vs. NM_WW, HX-3 vs. NM_WW, WG-1 vs. NM_WW, WG-2 vs. NM_WW, as well as WG-3 vs. NM_WW). (TIF)

S4 Fig The Person correlation analysis based on log2 fold change measurement of the 15 putative DEGs related to anthocyanin synthesis for the qRT-PCR analysis is higher between RNA-Seq and qRT-PCR. (TIF)

S5 Fig Venn diagram analysis of common anthocyanin synthesis related genes in seven different fleshy root libraries (NM_WW, HX-1, HX-2, HX-3, WG-1, WG-2 and WG-3) (HX-1 VS NM_WW, HX-2 VS NM_WW, HX-3 VS NM_WW, WG-1 VS NM_WW, WG-2 VS NM_WW, as well as WG-3 VS NM_WW). (TIF)

S1 Table Characteristics of the selected materials and their corresponding pigment content. (XLSX)

S2 Table List of primers used for qRT-PCR analysis of anthocyanin synthesis-related genes (ASRGs) identified in radish. (XLSX)

S3 Table Preprocess of RNA-Seq data obtained from each different radish fleshy roots. (XLSX)

S4 Table The abundance of globally expressed genes identified using FRKM in seven different radish fleshy root types. (XLSX)

S5 Table Expression levels of co-modulated DEGs identified in seven different radish fleshy root types. (XLSX)

S6 Table Relative expression levels of candidate anthocyanin synthesis-related genes (ASRGs) identified in radish. (XLSX)

S7 Table GO annotation of co-modulated DEGs in radish. (XLSX)

S8 Table KEGG pathway enrichment of co-modulated DEGs in radish. (XLSX)

## References

[1] Khoo HE, Azlan A, Tang ST, Lim SM. Anthocyanidins and anthocyanins: colored pigments as food, pharmaceutical ingredients, and the potential health benefits. Food Nutr Res.2017; 61(1): 1361779 10.1080/16546628.2017.1361779 28970777 PMC5613902

[2] Gul K. Health benefits of anthocyanins and their encapsulation for potential use in food systems: a review. Crit Rev Food Sci Nutr. 2016; 56(6): 2223–2230. 10.1080/10408398.2013.805316 25745811

[3] He J, Giusti M. Anthocyanins: Natural colorants with health-promoting properties. Annu Rev Food Sci Technol. 2010;1: 163 10.1146/annurev.food.080708.100754 22129334

[4] Bajpai A.; Khan K.; Muthukumar M.; Rajan S.; Singh N.K. Molecular analysis of anthocyanin biosynthesis pathway genes and their differential expression in mango peel. Genome. 2018; 61(3): 157–166. 10.1139/gen-2017-0205 29338343

[5] Aza-González C, Herrera-Isidrón L, Núñez-Palenius HG, Vega OMDL, Ochoa-Alejo N. Anthocyanin accumulation and expression analysis of biosynthesis-related genes during chili pepper fruit development. Biol Plantarum. 2013; 57(1): 49–55. 10.1007/s10535-012-0265-1

[6] Dao TTH, Linthorst HJM, Verpoorte R. Chalcone synthase and its functions in plant resistance. Phytochem Rev. 2011; 10(3): 397–412. 10.1007/s11101-011-9211-7 21909286 PMC3148432

[7] Azuma A. Genetic and environmental impacts on the biosynthesis of anthocyanins in grapes. Horticult J. 2018; 87(3): 1–17.

[8] Crifò T, Puglisi I, Petrone G, Recupero GR, Lo Piero AR. Expression analysis in response to low temperature stress in blood oranges: implication of the flavonoid biosynthetic pathway. Gene. 2011; 476(1–2): 1–9. 10.1016/j.gene.2011.02.005 21349317

[9] Li XY, Sun HY, Pei JB, Dong YY, Wang FW, Chen H, et al *De novo* sequencing and comparative analysis of the blueberry transcriptome to discover putative genes related to antioxidants. Gene. 2012; 511(1): 54–61. 10.1016/j.gene.2012.09.021 22995346

[10] Quast C, Pruessel E, Yilmaz P, Gerken J, Schweer T, Yarza P, et al The SILVA ribosomal RNA gene database project: improved data processing and web-based tools. Nucleic Acids Res. 2013; 41(Database issue): 590–596. 10.1093/nar/gks1219 23193283 PMC3531112

[11] Li H, Durbin R. Fast and accurate short read alignment with Burrows-Wheeler transform. Bioinformatics. 2009; 25(14): 1754–1760. 10.1093/bioinformatics/btp324 19451168 PMC2705234

[12] Li B, Dewey CN. RSEM: accurate transcript quantification from RNA-Seq data with or without a reference genome. BMC Bioinformatics. 2011; 12: 323 10.1186/1471-2105-12-323 21816040 PMC3163565

[13] Tarazona S, García F, Ferrer A, Dopazo J, Conesa A. NOIseq: a RNA-Seq differential expression method robust for sequencing depth biases. EMB Net J. 2012; 17(): 18–19. https://10.14806/ej.17.B.265

[14] Xie C, Mao XZ, Huang JJ, Ding Y, Wu JM, Dong S, et al KOBAS 2.0: a web server for annotation and identification of enriched pathways and diseases. Nucleic acids Res. 2011; 39(Web Server issue): 316–322. 10.1093/nar/gkr483 21715386 PMC3125809

[15] Gao J, Luo M, Zhang C, Peng H, Lin HJ, Shen YO, et al A putative pathogen-resistant regulatory pathway between MicroRNAs and candidate target genes in maize. J Plant Biol. 2015; 58(4): 211–219. https://10.1007/s12374-014-0572-5

[16] Schefe JH, Lehmann KE, Buschmann IR, Unger T, Funke-Kaiser H. Quantitative real-time RT-PCR data analysis: current concepts and the novel “gene expression’s C T difference” formula. J Mol Med. 2006; 84(11): 901–910. 10.1007/s00109-006-0097-6 16972087

[17] Liu YL, Che F, Wang LX, Meng R, Zhang XJ, Zhao ZY. Fruit coloration and anthocyanin biosynthesis after bag removal in non-red and red apples (*Malus* × *domestica Borkh*.). Molecules. 2013; 18(2): 1549–1563. 10.3390/molecules18021549 23353125 PMC6269864

[18] Soubeyrand E, Basteau C, Hilbert G, van Leeuwen C, Delrot S, Gomès E. Nitrogen supply affects anthocyanin biosynthetic and regulatory genes in grapevine cv. Cabernet-Sauvignon berries. Phytochemistry. 2014; 103: 38–49. 10.1016/j.phytochem.2014.03.024 24735825

[19] Zhao J, Dixon RA. The 'ins' and 'outs' of flavonoid transport. Trends Plant Sci. 2010; 15(2): 72–80. 10.1016/j.tplants.2009.11.006 20006535

[20] Marrs KA, Alfenito MR, Lloyd AM, Walbot VA. Glutathione S-transferase involved in vacuolar transfer encoded by the maize gene Bronze-2. Nature. 1995; 375(6530): 397–400. 10.1038/375397a0 7760932

[21] Kitamura S, Shikazono N, Tanaka A. TRANSPARENT TESTA 19 is involved in the accumulation of both anthocyanins and proanthocyanidins in Arabidopsis. Plant J. 2004; 37(1): 104–114. 10.1046/j.1365-313x.2003.01943.x 14675436

[22] Espley RV, Brendolise C, Chagne D, Kutty-Amma S, Green S, Volz R, et al Multiple repeats of a promoter segment causes transcription factor autoregulation in red apples. Plant Cell. 2009; 21(1): 168–183. 10.1105/tpc.108.059329 19151225 PMC2648084

[23] Hoffmann T, Kalinowski G, Schwab W. RNAi-induced silencing of gene expression in strawberry fruit *(Fragaria × ananassa)* by agroinfiltration: a rapid assay for gene function analysis. Plant J. 2006; 48(5): 818–826. 10.1111/j.1365-313X.2006.02913.x 17092319

[24] Jiang F, Wang JY, Jia HF, Jia WS, Wang HQ, Xiao M. RNAi-Mediated silencing of the flavanone 3-hydroxylase gene and its effect on flavonoid biosynthesis in strawberry fruit. J Plant Growth Regul. 2013; 32(1): 182–190. https://10.1007/s00344-012-9289-1

[25] Lin X, Xiao M, Luo Y, Wang J, Wang H. The effect of RNAi-induced silencing of FaDFR on anthocyanin metabolism in strawberry (*Fragaria × ananassa*) fruit. Sci Hortic-Amsterdam. 2013; 160:123–128. https://10.1016/j.scienta.2013.05.024

[26] Dixon RA, Liu C, Jun JH. Metabolic engineering of anthocyanins and condensed tannins in plants. Curr Opin Biotech. 2013; 24(2): 329–335. 10.1016/j.copbio.2012.07.004 22901316

[27] Li S. Transcriptional control of flavonoid biosynthesis: fine-tuning of the *myb-bhlh-wd40* (mbw) complex. Plant Signal. Behav. 2014, 9(1): e27522 10.4161/psb.27522 24393776 PMC4091223

