# Supplementary figures and images for: Identification of differential expression genes related to anthocyanin biosynthesis in carmine radish (Raphanus sativus L.) fleshy roots using comparative RNA-Seq method
Source: PLoS One. 2020 Apr 24;15(4):e0231729. doi: 10.1371/journal.pone.0231729 (PMC7182184; doi:10.1371/journal.pone.0231729)

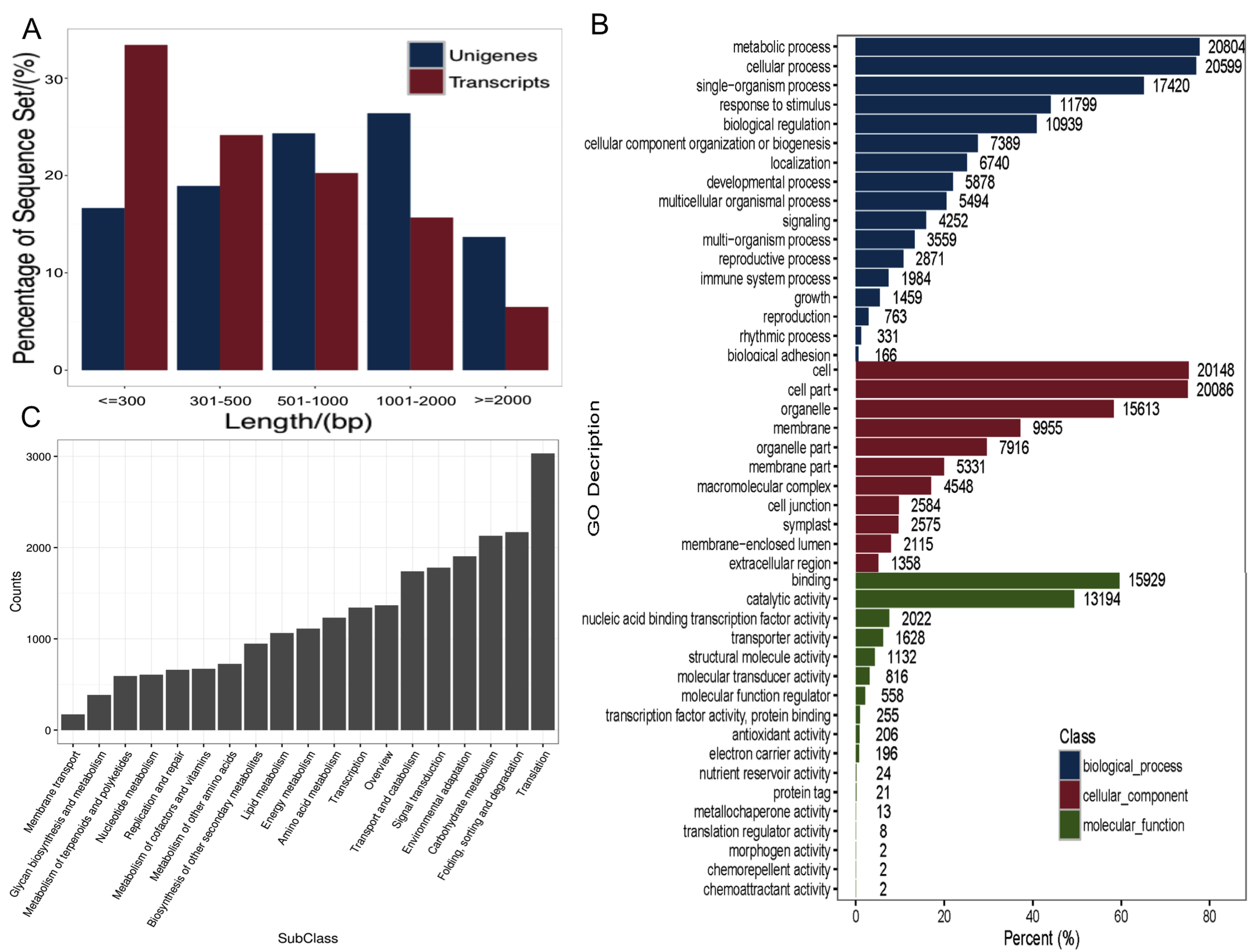

Supplement: S1 Fig — A. Length distribution of contigs and unigenes from radish transcriptome, B. Histogram representation of GO terms. Unigenes were assigned to gene othology (GO) terms comprising “BP”, “MF,” and “CC” for functional classification. C. Pathway assignment based on the KEGG, Non-redundant unigenes for radish transcriptome were assigned into 138 biosynthesis pathways and classified into 19 subclass categories. (TIF) [file pone.0231729.s001.tif]

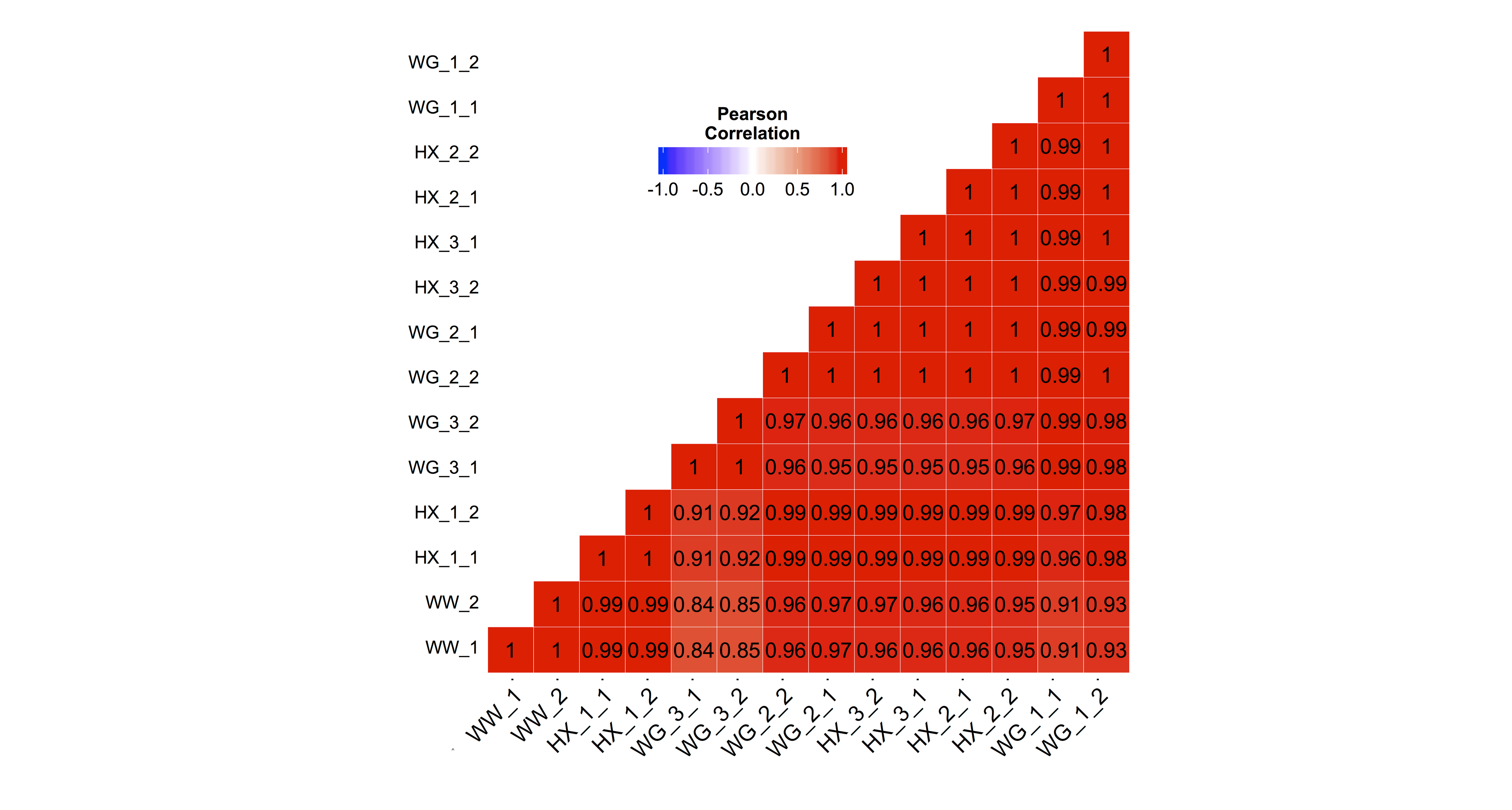

Supplement: S2 Fig — The hierarchical clustering dendrogram was inferred according to SCC analysis result. (TIF) [file pone.0231729.s002.tif]

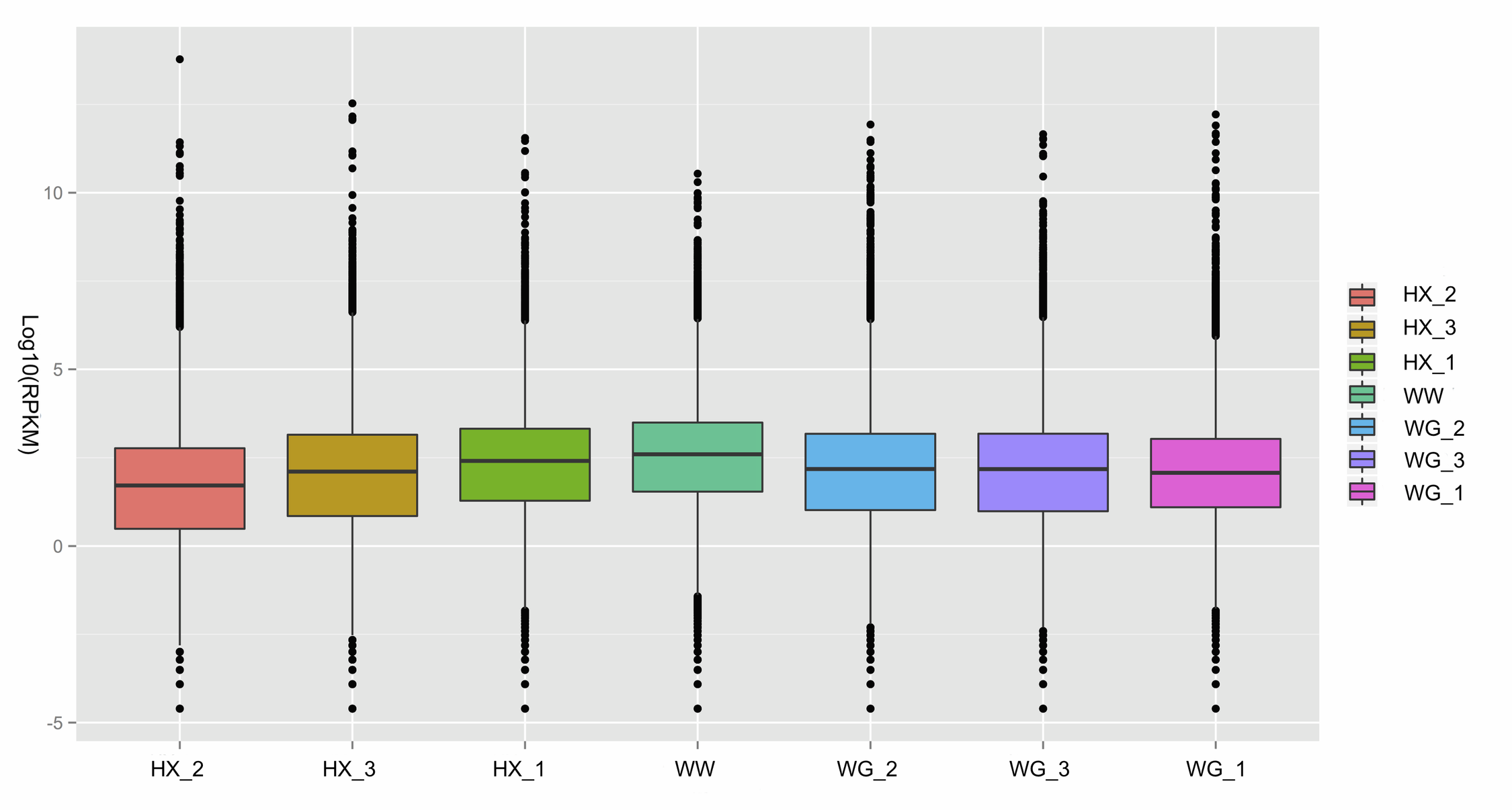

Supplement: S3 Fig — (TIF) [file pone.0231729.s003.tif]

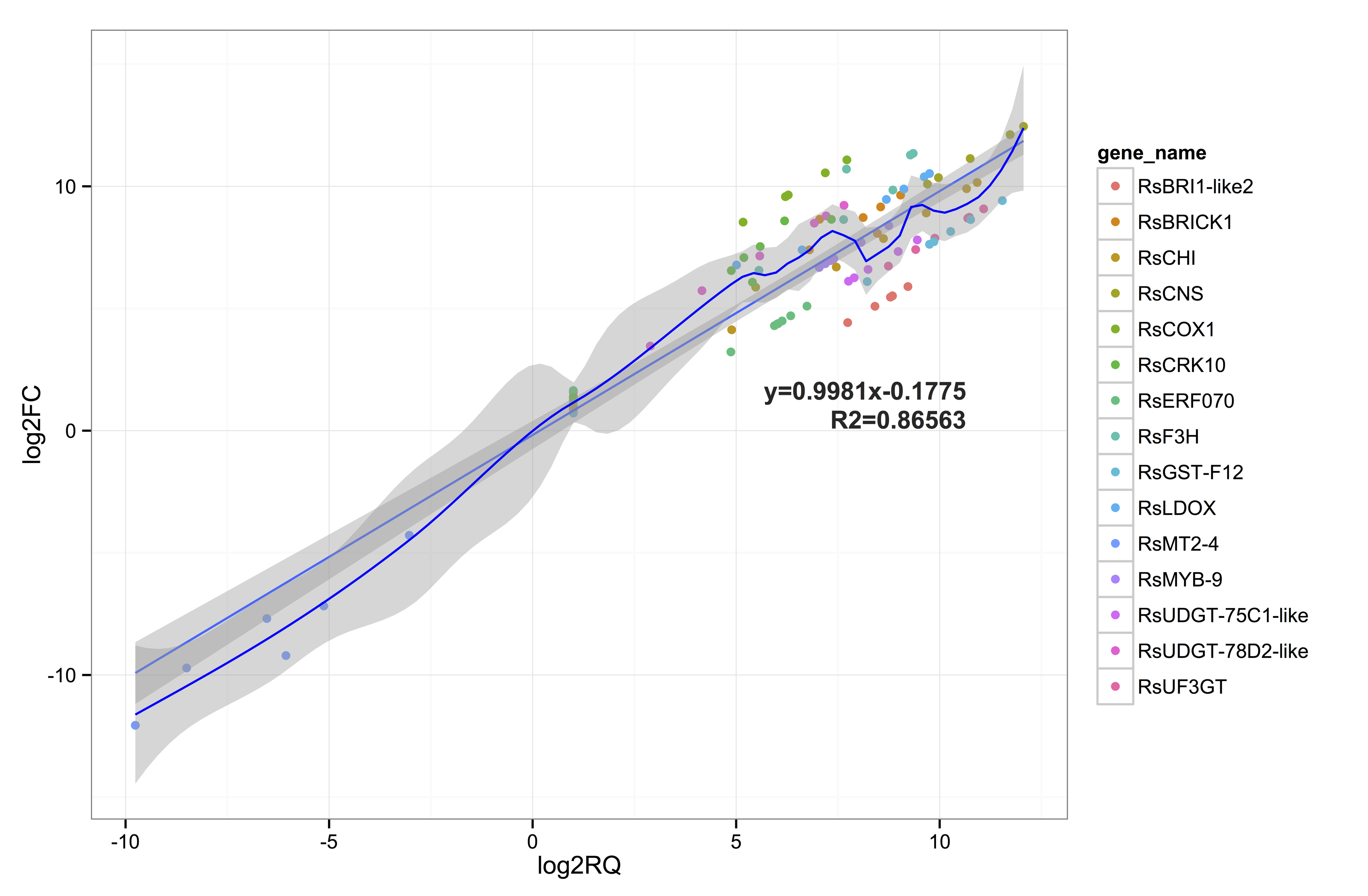

Supplement: S4 Fig — (TIF) [file pone.0231729.s004.tif]

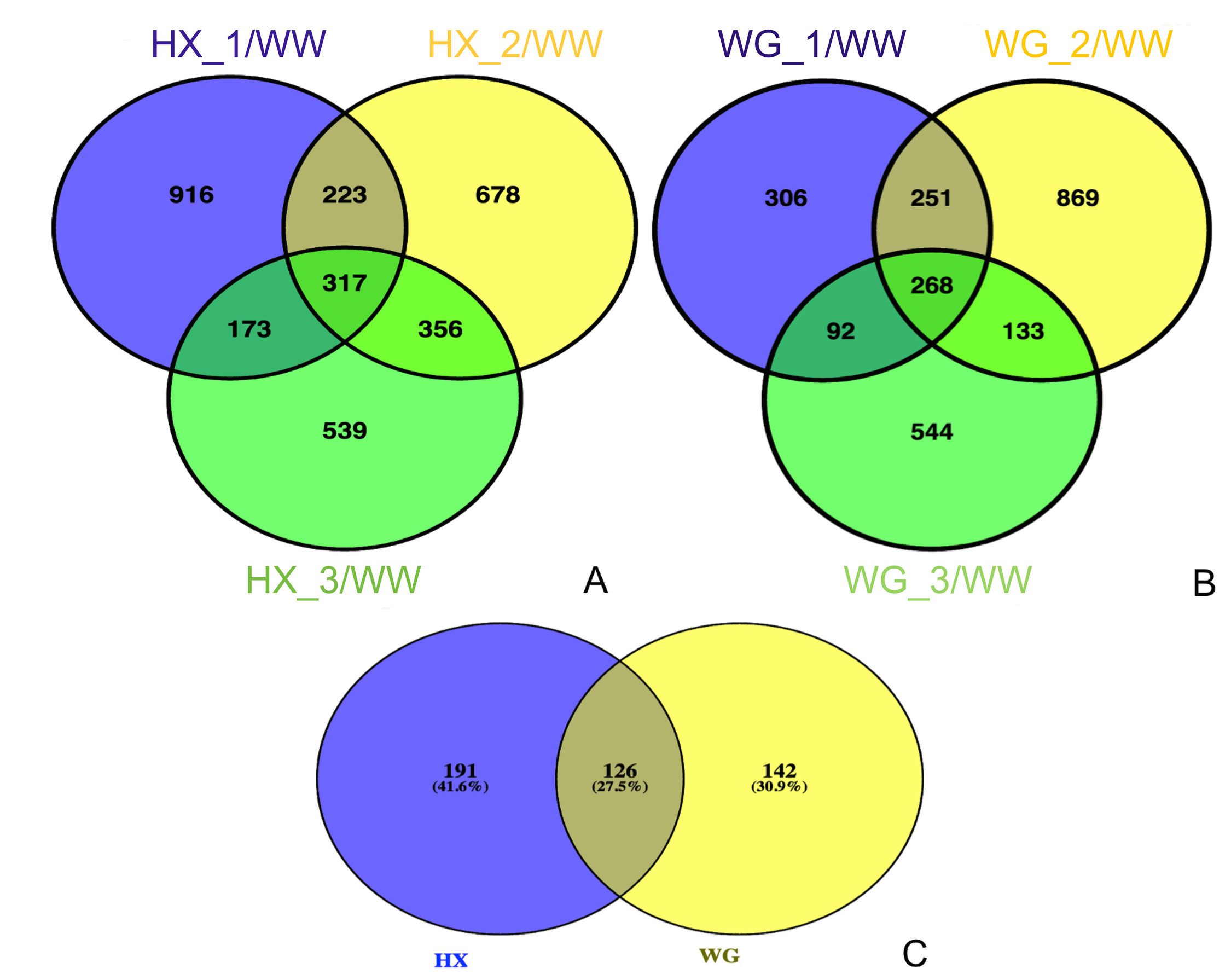

Supplement: S5 Fig — (TIF) [file pone.0231729.s005.tif]
